# Scaling properties of ballistic nano-transistors

**DOI:** 10.1186/1556-276X-6-365

**Published:** 2011-04-28

**Authors:** Ulrich Wulf, Marcus Krahlisch, Hans Richter

**Affiliations:** 1 BTU Cottbus, Fakultät 1, Postfach 101344, 03013 Cottbus, Germany

## Abstract

Recently, we have suggested a scale-invariant model for a nano-transistor. In agreement with experiments a close-to-linear thresh-old trace was found in the calculated *I*_D _- *V*_D_-traces separating the regimes of classically allowed transport and tunneling transport. In this conference contribution, the relevant physical quantities in our model and its range of applicability are discussed in more detail. Extending the temperature range of our studies it is shown that a close-to-linear thresh-old trace results at room temperatures as well. In qualitative agreement with the experiments the *I*_D _- *V*_G_-traces for small drain voltages show thermally activated transport below the threshold gate voltage. In contrast, at large drain voltages the gate-voltage dependence is weaker. As can be expected in our relatively simple model, the theoretical drain current is larger than the experimental one by a little less than a decade.

## Introduction

In the past years, channel lengths of field-effect transistors in integrated circuits were reduced to arrive at currently about 40 nm [1]. Smaller conventional transistors have been built [2-9] with gate lengths down to 10 nm and below. As well-known with decreasing channel length the desired long-channel behavior of a transistor is degraded by short-channel effects [10-12]. One major source of these short-channel effects is the multi-dimensional nature of the electro-static field which causes a reduction of the gate voltage control over the electron channel. A second source is the advent of quantum transport. The most obvious quantum short-channel effect is the formation of a source-drain tunneling regime below threshold gate voltage. Here, the *I*_D _- *V*_D_-traces show a positive bending as opposed to the negative bending resulting for classically allowed transport [13,14]. The source-drain tunneling and the classically allowed transport regime are separated by a close-to linear threshold trace (LTT). Such a behavior is found in numerous MOSFETs with channel lengths in the range of a few tens of nanometers (see, for example, [2-9]).

Starting from a three-dimensional formulation of the transport problem it is possible to construct a one-dimensional effective model [14] which allows to derive scale-invariant expressions for the drain current [15,16]. Here, the quantity  arises as a natural scaling length for quantum transport where *ε*_F _is the Fermi energy in the source contact and m* is the effective mass of the charge carriers. The quantum short-channel effects were studied as a function of the dimensionless characteristic length *l *= *L/λ *of the transistor channel, where *L *is its physical length.

In this conference contribution, we discuss the physics of the major quantities in our scale-invariant model which are the chemical potential, the supply function, and the scale-invariant current transmission. We specify its range of applicability: generally, for a channel length up to a few tens of nanometers a LTT is definable up to room temperature. For higher temperatures, a LTT can only be found below a channel length of 10 nm. An inspection of the *I*_D _- *V*_G_-traces yields in qualitative agreement with experiments that at low drain voltages transport becomes thermally activated below the threshold gate voltage while it does not for large drain voltages. Though our model reproduces interesting qualitative features of the experiments it fails to provide a quantitative description: the theoretical values are larger than the experimental ones by a little less than a decade. Such a finding is expected for our simple model.

## Theory

### Tsu-Esaki formula for the drain current

**Figure 1:**
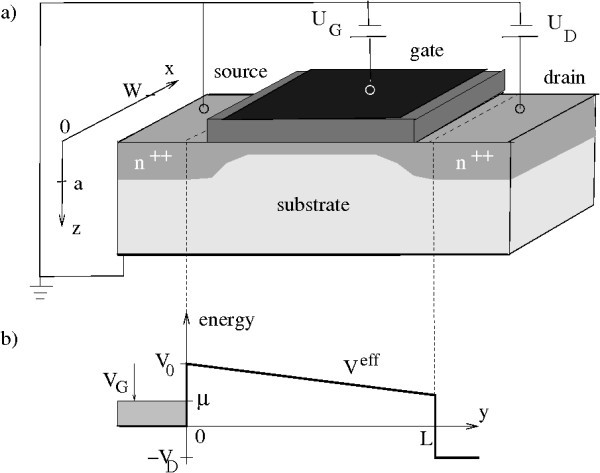
**Generic n-channel nano-field effect transistor**. **(a) **Schematic representation. **(b) **One-dimensional effective potential *V*^eff^.

In Refs. [13,14], the transport problem in a nano-FET was reduced to a one-dimensional effective problem invoking a "single-mode abrupt transition" approximation. Here, the electrons move along the transport direction in an effective potential given(1)

(see Figure 1b). The energy zero in Equation 1 coincides with the position of the conduction band minimum in the highly n-doped source contact. As shown in [14](2)

where *E*_*k *= 1 _is the bottom of the lowest two-dimensional subband resulting in the z-confinement potential of the electron channel at zero drain voltage (see Figure 4b of Ref. [13]). The parameter *W *is the width of the transistor. Finally, *V*_D _= *eU*_D _is the drain potential at drain voltage *U*_D _which is assumed to fall off linearly.

Experimentally, one measures in a wide transistor the current density *J*, which is the current per width of the transistor that we express as(3)

Here  is the number of equivalent conduction band minima ('valleys') in the electron channel and *I*_0 _= 2*eε*_F_/*h*. In Refs. [15,16] a scale-invariant expression(4)

was derived. Here, *m *= *μ*/*ε*_F _is the normalized chemical potential in the source contact, *v*_D _= *V*_D_/*ε*_F _is the normalized drain voltage, and *v*_G _= *V*_G_/*ε*_F _is the normalized gate voltage. As illustrated in Figure 1(b) the gate voltage is defined as the energy difference *μ *- *V*_0 _= *V*_G_, i.e., for *V*_G _> 0 the transistor operates in the ON-state regime of classically allowed transport and for *V*_G _< 0 in the source-drain tunneling regime. The control variable *V*_G _is used to eliminate the unknown variable *V*_0_. For the chemical potential in the source contact one finds (see next section)(5)

where *u *= *k_B_T*/*ε*_F _is the normalized thermal energy. Equation 4 has the form of a Tsu-Esaki formula with the normalized supply function(6)

Here, *F*_-1/2 _is the Fermi-Dirac integral  of order -1/2 and  is the inverse function of *F*_1/2_. The effective current transmission  depends on  which is the normalized energy of the electron motion in the *y*-*z*-plane while  is their energy in the *x*-direction. In the next sections, we will discuss the occurring quantities in detail.

### Chemical potential in source- and drain-contact

For a wide enough transistor and a sufficient junction depth *a *(see Figure 1) the electrons in the contacts can be treated as a three-dimensional non-interacting electron gas. Furthermore, we assume that all donor impurities of density *N_i _*are ionized. From charge neutrality it is then obtained that the electron density *n*_0 _is independent of the temperature and given by(7)

Here *m*^e ^is the effective mass and *N*_V _is the valley-degeneracy factor in the contacts, respectively. In the zero temperature limit a Sommerfeld expansion of the Fermi-Dirac integral leads to(8)

**Figure 2:**
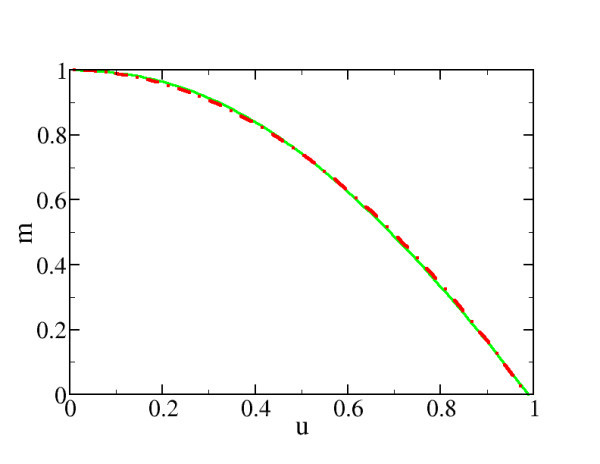
**Normalized chemical potential vs. thermal energy according to Equation 9 in green solid line and parabolic approximation in red dash-dotted line**.

Equating 7 and 8 results in(9)

which is identical with (5) and plotted in Figure 2. As well-known, with increasing temperature the chemical potential falls off because the high-energy tail of the Fermi-distribution reaches up to ever higher energies.

### Supply function

As shown in Ref. [14] the supply function for a wide transistor can be written as(10)

This expression can be interpreted as the partition function (loosely speaking the "number of occupied states") in the grand canonic ensemble of a non-interacting homogeneous three-dimensional electron gas in the subsystem of electrons with a given lateral wave vector (*k_y_*, *k_z_*) yielding the energy  in the *y*-*z*-direction. Formally equivalent it can be interpreted as the full partition function in the grand canonic ensemble of a one-dimensional electron gas at the chemical potential *μ *- *ε*. Performing the limit  the Riemann sum in the variable  can be replaced by the Fermi-Dirac integral *F*_-1/2_. It results that(11)

with the normalized transistor width *w *= *W*/*λ*. For the scaling of the supply function in Equation 11 we define (see Ref. [14])(12)

where  and we use the identity *V*_0_= *ε*_F _= *m *- *v*_G_. For the source contact we write(13)

leading to the first factor in the square bracket of the Tsu-Esaki equation 4. In the drain contact, the chemical potential is lower by the factor *V*_D_. Replacing *μ *→ *μ *- *V*_D _yields(14)

Below we will show that for transistor operation the low temperature limit is relevant (see Figure 2). Here, one may apply in leading order  (resulting from a Sommerfeld expansion) and *F*_-1/2_(-*x *→ ∞) → exp (*x*). Since *V*_0 _> 0 the factor *v*_G _- *m *is negative and we obtain from (12)(15)

**Figure 3:**
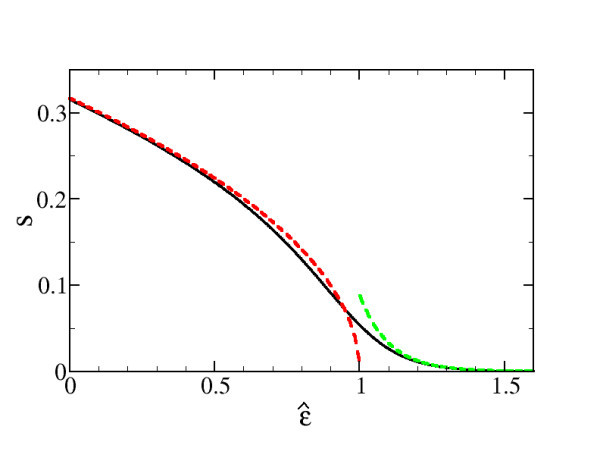
**Supply function in the source contact (see Equation 6) for *u *= 0.1 and *v*_G _= 0 (black line), low-temperature limit according to Equation 15 for *α *< 0 (red dashed line) and *α *> 0 (green dashed line)**. Because of the small temperature *m*(*u*) ~ 1 so that  occurs at .

From Figure 3 it is seen that for *ε *below the chemical potential the supply function is well described by the square-root dependence in the  limit. If *ε *lies above the chemical chemical one obtains the  limit which is a small exponential tail due to thermal activation.

### Current transmission

The effective current transmission in Equation 16 is given y(16)

It is calculated from the scattering solutions of the scaled one-dimensional Schrödinger equation(17)

with *β *= 2m*V_0_*L*^2^/*ħ*^2 ^= *l*^2^(*m *- *v*_G_), and *ŷ *= *y*/*L*. The scaled effective potential  is given by , , and ,where . As usual, the scattering functions emitted from the source contact  obey the asymptotic conditions  and(18)

with  and .

**Figure 4:**
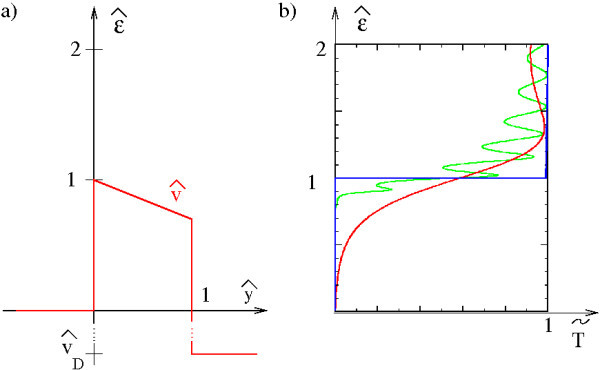
**Scaled effective model**. **(a) **Scaled effective potential. **(b) **Effective current transmission at *u *= 0.1, *v*_D _= 0.5, and *v*_G _= 0 ( = 0.504 and *m *= 0.992). The considered characteristic lengths are *l *= 4 (red, weak barrier, *β *= 15.87) and *l *= 25 (green, strong barrier, *β *= 619.8). The ideal limit (Equation 19) in blue line.

As can be seen from Figure 4, around  the current transmission changes from around zero to around one. For weak barriers there is a relatively large current transmission below one leading to drain leakage currents. For strong barriers this remnant transmission vanishes and we can approximate the current transmission by an ideal one.(19)

To a large extent the Fowler Nordheim oscillations in the numerical transmission average out performing the integration in Equation 4.

### Parameters in experimental nano-FETs

#### Heavily doped contacts

In the heavily doped contacts the electrons can be approximated as a three-dimensional non-interacting Fermi gas. Then from (8) the Fermi energy above the bottom of the conduction band is given by(20)

For *n*^++^-doped Si contacts the valley-degeneracy is *N*_V _= 6 and the effective mass is taken as . Here *m*_1 _= 0.19*m*_0 _and *m*_2 _= 0.98*m*_0 _are the effective masses corresponding to the principle axes of the constant energy ellipsoids. In our later numerical calculations we set *ε*_F _= 0.35 eV assuming a level of source-doping as high as *N*_i _= *n*_0 _= 10^21 ^cm^-3^.

#### Electron channel

In the electron channel a strong lateral subband quantization exists As well-known [17] at low temperatures only the two constant energy ellipsoids with the heavy mass *m*_2 _perpendicular to the (100)-interface are occupied leading to a valley degeneracy of *g*_v _= 2. The in-plane effective mass is therefore the light mass m* = *m*_1 _entering the relation(21)

Here *ε*_F _= 0.35 eV was assumed. One then has in Equation 3 *I*_0 _= ~ 27*μ*A and with *λ *~ 1 nm as well as  = 2 one obtains *J*_0 _= 5.4 × 10^4 ^*μ*A/*μ*m.

## Results

### Drain characteristics

**Figure 5:**
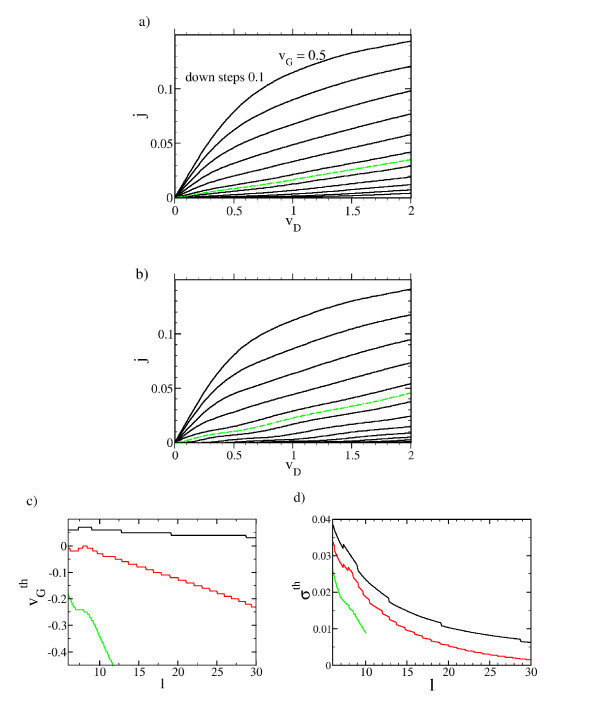
**Calculated drain characteristics for *l *= 10, *v_G _*starting from 0.5 with decrements of 0.1 (solid lines) at the temperature (a) *u *= 0.1 and (b) *u *= 0.01**. In green dashed lines the LTT. For *u *= 0.1 the LTT occurs at a gate voltage of  = -0.05 and for *u *= 0.01 at  = 0.05. **(c) **, and **(d) **σ^th ^versus characteristic length for *u *= 0.01 (black), *u *= 0.1 (red), and *u *= 0.2 (green).

Typical drain characteristics are plotted in Figure 5 for a low temperature (*u *= 0.01) and at room temperature (*u *= 0.1). It is seen that for both the temperatures a LTT can be identified. We define the LTT as the *j *- *v*_D _trace which can be best fitted with a linear regression *j *= σ^th^*v*_D _in the given interval 0 ≤ *v*_D _≤ 2. The best fit is determined by the minimum relative mean square deviation. The gate voltage associated with the LTT is denoted with . It turns out that at room temperature  lies slightly above zero and at low temperatures slightly below (see Figure 5c). In general, the temperature dependence of the drain current is small. The most significant temperature effect is the enhancement of the resonant Fowler-Nordheim oscillations found at negative *v*_G _at low temperatures. From Figure 5d, it can be taken that the slope of the LTT σ^th ^decreases with increasing *l *and increasing temperature. For "hot" transistors (*u *= 0.2) a LTT can only be defined up to *l *~ 10.

### Threshold characteristics

**Figure 6:**
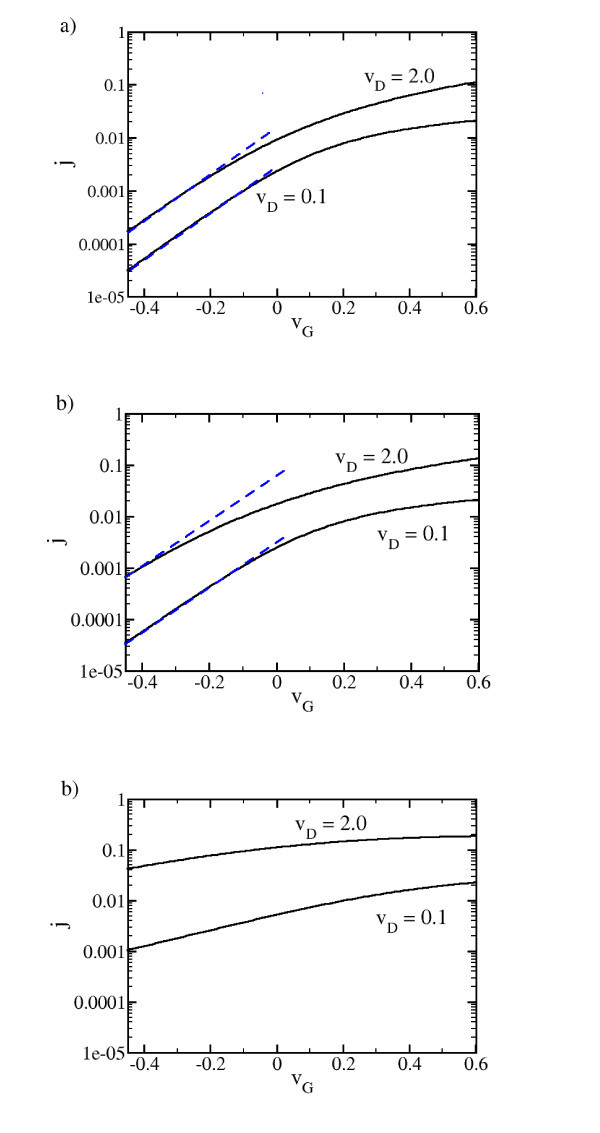
**Calculated threshold characteristics at *u *= 0.1 (a) for *l *= 60 and (b) *l *= 25, and (c) *l *= 3**. The dashed straight lines in blue are guides to the eye exhibiting a slope corresponding to thermal activation.

The threshold characteristics at room temperature are plotted in Figure 6 for a "small" drain voltage (*v*_D _= 0.1) and a "large" drain voltage (*v*_D _= 2.0). For the largest considered characteristic length *l *= 60 it is seen that below zero gate voltage the drain current is thermally activated for both considered drain voltages. A comparison with the results for *l *= 25 and *l *= 10 yields that for the small drain voltage the *I*_D _- *V*_G _trace is only weakly effected by the change in the barrier strength. In contrast, at the high drain voltage the drain current below *v*_G _= 0 grows strongly with decreasing barrier strength. The drain current does not reach the thermal activation regime any more, it falls of much smoother with increasing negative *v*_G_. As can be gathered from Figure 8 this effect is seen in experiments as well. We attribute it to the weakening of the tunneling barrier with increasing *v*_D_. To confirm this point the threshold characteristics for a still weaker barrier strength (*l *= 3) is considered. No thermal activation is found in this case even for the small drain voltage.

## Discussion

**Figure 7:**
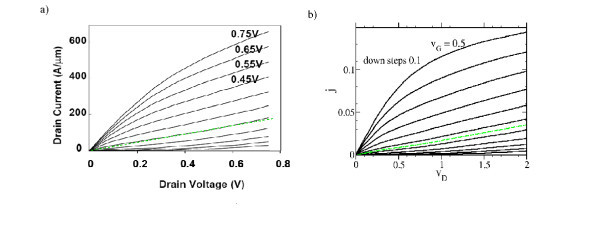
**Drain characteristics in experiment and theory**. **(a) **Experimental drain characteristics for a nano-transistor with *L *= 10 nm [4,5]. Our assumption for the LTTis marked with a green dashed line leading to a threshold gate voltage of  = 0.15V. **(b) **Theoretical drain characteristics for *l *= 10 and *u *= 0.1 (see Fig. 5a) with the green dashed threshold characteristic at  = -0.05.

We discuss our numerical results on the background of experimental characteristics for a 10 nm gate length transistor [4,5] reproduced in Figure 7. As demonstrated in Sect. "Parameters in experimental nano-FETs" one obtains from Equation 21 a characteristic length of *λ *~ 1 nm under reasonable assumptions. For the experimental 10 nm gate length, we thus obtain *l *= *L*/*λ *= 10. Furthermore, Equation 20 yields the value of *ε*_F _= 0.35 eV. The conversion of the experimental drain voltage *V *into the theoretical parameter *v*_D _is given by(22)

**Figure 8:**
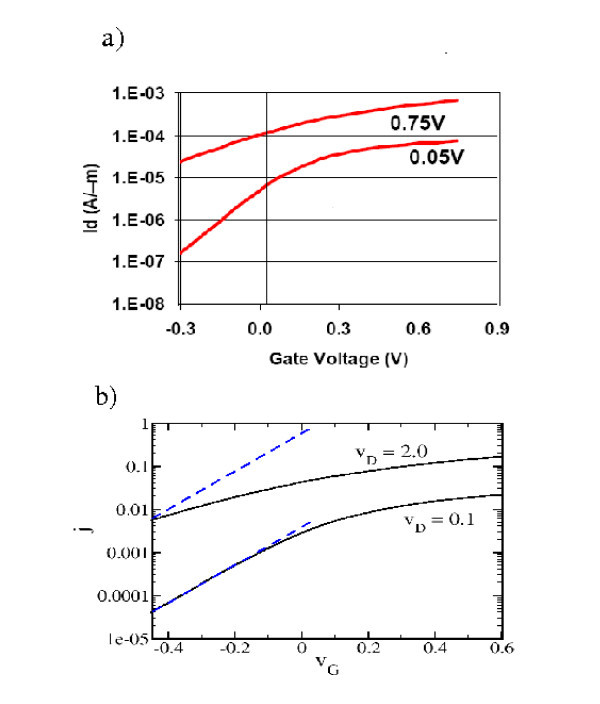
**Threshold characteristics in experiment and theory**. **(a) **Experimental threshold characteristics for the nano-transistor in Fig. 7a. **(b) **Theoretical threshold characteristics for *l *= 10 and *u *= 0.1 with the blue dashed lines corresponding to thermal activation.

The maximum experimental drain voltage of 0.75 V then sets the scale for *v*_D _ranging from zero to *v*_D _= 0.75 eV/0.35 eV ~ 2. For the conversion experimental gate voltage *V*_G _to the theoretical parameter *v*_G _we make linear ansatz as(23)

where  is the experimental threshold gate voltage (see Figure 8a). The constant *β *is chosen so that  converts into . In our example, it is shown from Figure 8a = 0.15 V and from Figure 8b = -0.05, so that *β *= -0.2 eV. To match the experimental drain characteristic to the theoretical one we first convert the highest experimental value for *V*_G _into the corresponding theoretical one. Inserting in (23) *V*_G _= 0.75 V yields *v*_G _~ 0.5. Second, we adjust the experimental and the theoretical drain current-scales so that in Figure 7 the curves for the experimental current at *V*_G _= 0.7 and the theoretical curve at *v*_G _= 0.5 agree. It then turns out that the other corresponding experimental and theoretical traces agree as well. This agreement carries over to the range of negative gate voltages with thermally activated transport. This can be gathered from the *I*_D _- *V*_G _traces in Figure 8. We note that the constant of proportionality in Equation 23 given by 1 eV is more then *ε*_F _which one would expect from the theoretical definition *v*_G _= *V*_G_/*ε*_F_. Here, we emphasize that the experimental value of *e V*_G _corresponds to the change of the potential at the transistor gate while the parameter *v*_G _describes the position of the bottom of the lowest two-dimensional subband in the electron channel. The linear ansatz in Equation 23 and especially the constant of proportionality 1 eV can thus only be justified in a self-consistent calculation of the subband levels as has been provided, e.g., by Stern[18].

The experimental and the theoretical drain characteristics in Figure 7 look structurally very similar. For a quantitative comparison we recall from Sect. "Parameters in experimental nano-FETs" the value of *J*_0 _= 5.4 × 10^4^*μ*A/*μ*m. Then the maximum value *j *= 0.15 in Figure 7b corresponds to a theoretical current per width of 8 × 10^3^*μ*A/*μ*m. To compare with the experimental current per width we assume that in the *y*-axis labels in Figures 7a and 8a it should read *μ*A/*μ*m instead of A/*μ*m. The former unit is the usual one in the literature on comparable nanotransistors (see Refs. [2-9]) and with this correction the order of magnitude of the drain current per width agrees with that of the comparable transistors. It is found that the theoretical results are larger than the experimental ones by about a factor of ten. Such a failure has to be expected given the simplicity of our model. First, for an improvement it is necessary to proceed from potentials resulting in a self-consistent calculation. Second, our representation of the transistor by an effectively one-dimensional system probably underestimates the backscattering caused by the relatively abrupt transition between contacts and electron channel. Third, the drain current in a real transistor is reduced by impurity interaction, in particular, by inelastic scattering. As a final remark we note that in transistors with a gate length in the micrometer scale short-channel effects may occur which are structurally similar to the ones discussed in this article (see Sect. 8.4 of [10]). Therefore, a quantitatively more reliable quantum calculation would be desirable allowing to distinguish between the short-channel effects on micrometer scale and quantum short-channel effects.

## Summary

After a detailed discussion of the physical quantities in our scale-invariant model we show that a LTT is present not only in the low temperature limit but also at room temperatures. In qualitative agreement with the experiments the *I*_D _- *V*_G_-traces exhibit below the threshold voltage thermally activated transport at small drain voltages. At large drain voltages the gate-voltage dependence of the traces is much weaker. It is found that the theoretical drain current is larger than the experimental one by a little less than a decade. Such a finding is expected for our simple model.

## Abbreviation

LTT: linear threshold trace.

## Competing interests

The authors declare that they have no competing interests.

## Authors' contributions

UW worked out the theroretical model, carried out numerical calculations and drafted the manuscript. MK carried out numerical calculations and drafted the manuscript. HR drafted the manuscript. All authors read and approved the final manuscript.
